# Paederus Dermatitis Outbreak in Addis Ababa, Ethiopia: A Case-Control Study

**DOI:** 10.1155/2021/8892785

**Published:** 2021-03-15

**Authors:** G. Neamin, A. Negga, H. Mukemil, B. Mengistu, Y. Rahel

**Affiliations:** Center of Public Health Emergency Management, Ethiopian Public Health Institutes, Swaziland Street, P.O. Box 1242, Addis Ababa, Ethiopia

## Abstract

**Background:**

Paederus dermatitis is an irritant contact dermatitis caused by crushing insects of the genus *Paederus*, which releases a vesicant toxin called pederin. On July 28, 2018, the district health office received a report of cases with erythema, itching, and burning after contact with the *Paederus* insect. In response, we investigated the outbreak intending to describe, confirm, and identify the risk factors associated with the country's first reported outbreak.

**Methods:**

A community-based unmatched case-control study was conducted from August 10 to 22, 2018. Two hundred twenty-five (75 cases and 150 controls) study participants were involved in the study. Cases were defined as Bole subcity residents who had acute contact dermatitis after contact with the insect, while controls were persons who did not have contact with the insect. Cases were recruited consecutively as they present, whereas controls were selected by the neighborhood sampling method. An interviewer-administered questionnaire was used for the data collection, and multiple logistic regression was applied to determine the independent risk factors. The results were expressed as adjusted odds ratios (AORs) and 95% confidence intervals (CIs).

**Results:**

A total of 122 cases were reported from the three districts of the subcity. The mean age of cases and controls was 23.6 (SD ± 16.4) and 29.4 (SD ± 10.9) years, respectively. Multivariate analysis showed that the presence of outdoor light (AOR = 5.1; 95% CI (2.5, 10.9), presence of rotten leaves (AOR = 6.4; 95% CI (2.9, 15.7)), sleeping on the floor (AOR = 6.1; 95% CI (2.5, 15.7)), wearing protective clothing (AOR = 0.2; 95% CI (0.1, 0.4)), and use of insect repellant (AOR = 0.1; 95% CI (0.0, 0.4)) were significantly associated with Paederus dermatitis outbreak.

**Conclusion:**

The investigation identified exposure to artificial light and the presence of rotten leaves around the residential area as important factors leading to an increase in the odds of Paederus dermatitis. In contrast, the use of insect repellant and wearing protective clothing were shown to provide protection. The investigation determined that reducing burning outdoor lights, cleaning excess vegetation, avoiding sleeping on the floor, using insect repellants, and wearing protective clothing can reduce the risk of contracting Paederus dermatitis.

## 1. Introduction

Paederus dermatitis (PD) is an acute skin condition caused by exposure to a potent toxin called pederin. It is released when the beetles of the *Paederus* genus crushed. These beetles belong to the class Insecta, order Coleoptera (beetles), family Staphylinidae (rove beetles), subfamily Paederinae, tribe Paederini, and subtribe Paederina [1, 2]. Morphologically, *Paederus* beetles are relatively slender and uniquely identified by their size (1.5 mm width and 7–10 mm length) and coloring (usually bicolorous, black, or blue and red; sometimes, the entire body is black or blue or even reddish [3]. Typically, red or orange coloring is aposematic, warning predators that the beetle is toxic. The *Paederus* genus has approximately 650 species, 30 of which are shown to cause lineal dermatitis [4].

*Paederus* species are distributed worldwide and prefer moist habitats (e.g., under stones and on the banks of ponds, streams, rivers, and marshes). Adults are polyphagous and exhibit necrophagy and cannibalism. They feed mainly on other insects, moths, live tadpoles, and soil nematodes, decaying organic matter, but not on the living tissues of plants [5].

Pederin (C25H45O9N) is an amide with two tetrahydropyran rings and constitutes only 1% of the total insect body weight. Pederin is incredibly potent even in small concentrations, with the ability to inhibit cell growth at concentrations as low as 1.5 Nanograms per milliliter [2, 6]. The production of pederin is largely confined to the female insect [7]. Recently, it has been demonstrated that the production of pederin relies on the activities of an endosymbiont (*Pseudomonas* species) within the Beetle [8].

The clinical manifestation of PD may appear within 12–48 h after crushing of the insect on the skin [9, 10]. This acute irritant contact dermatitis is further characterized by burn-like lesions, which may be associated with vesicles, bullae, or pustules [11, 12]. In severe cases, erythema, blisters, and pigmented scars are more extensive and usually the result of crushing several Paederus onto the skin. In one outbreak attributed to the species *P. columbinus*, headache, fever, arthralgia, neuralgia, and nausea were reported [13, 14]. Additionally, epiphora and conjunctivitis have been reported in cases where the eye itself has been exposed to pederin [15]. There is also the possibility of secondary infection with residual pigmentation [5].

*Paederus* beetle populations increase rapidly during May and June. The peak time of rash during the year varied from one country to another. Commonly, the peak incidence of the disease is in May or April but may also occur in September or October [16].

Anjou-Kidoel lighthouse in Java, Indonesia, reported the first known outbreak in 1891 [17]. Since then, the two worst outbreaks were recorded in Okinawa in 1969 (nearly 2,000 cases) and Korea in 1995 (633 cases) [3]. Besides, the outbreaks of PD observed in Africa (Egypt, Sudan, Sierra Leone, Guinea, Nigeria, Central African Republic, Congo, Gabon, Namibia, Uganda, and Tanzania), Asia (Turkey, Iraq, Iran, Pakistan, India, Sri Lanka, China, Taiwan, Korea, and Okinawa), North and South America (USA, Venezuela, Brazil, and Ecuador), and Australia [3, 18]. However, in Ethiopia, the outbreak had not been reported previously. Therefore, we investigated the outbreak to describe, confirm, and identify the risk factors associated with the country's first reported outbreak.

## 2. Methods

### 2.1. Study Area and Period

Addis Ababa, the capital of Ethiopia, is the industrial, commercial, and cultural center of the country. Administratively, Addis Ababa is divided into ten subcities, and the subcities are further divided into 118 woredas (also called districts). Addis Ababa has a total population of 3,352,000, contributes to 4.3% and 40% of the inhabitant of the country and urban area, respectively, with a population density of 165.1 km^2^ and a total land area of 540 km^2^. In Addis Ababa, in 2016, there were a total of 92 public health centers and 11 public hospitals, with health centers to population ratio of 1 : 36435 and hospitals to population ratio of 1 : 304727 [19]. In 2017, acute respiratory infection, acute febrile illness, and pneumonia were the leading cause of morbidity, while prematurity, birth asphyxia, and neonatal sepsis were the leading causes of mortality [20].

**Figure 1:**
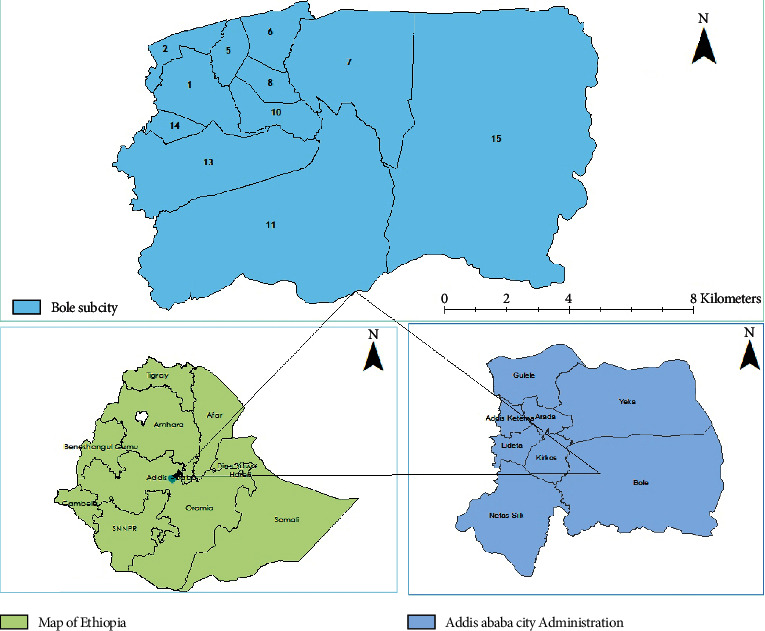
Map of Bole subcity with the respective district, Addis Ababa, Ethiopia, 2018.

Bole subcity is one of the largest subcities in Addis Ababa in terms of area hosting many business centers and shopping malls, organizations, recreational sites, and the Bole International Airport. The urban forest area coverage is relatively smaller than other subcities (Yeka, Gullele, Kolfe Keranyo, Nifas Silk-Lafto, and Akaki Kality) [21]. The actual study was conducted in three districts of the Bole subcity, namely, District-10, District-11, and District-15 (Figure 1). The investigation took place from July 10 to 22, 2018.

### 2.2. Study Design

We described the outbreak in person, place, and time using descriptive epidemiology to identify the possible exposures that were common to the cases. A community-based unmatched case-control study design was used to investigate the outbreak.

### 2.3. Case Definition and Inclusion and Exclusion Criteria

Cases were identified by a dermatologist using the following working case definition. “Any person with itching, burning sensation, blister, or any other lesion who have contact with PD causative beetles and resides in three districts of Bole subcity (10, 11, 15) during the study period.” A picture of the insect was shown to the cases to confirm contact for the final diagnosis. Cases were identified as individuals who fulfilled the above criteria. Case with a recent history of illness were included in the study.

### 2.4. Control Definition and Inclusion and Exclusion Criteria

Controls are identified as individuals who did not fulfill the above criteria and are selected from the same neighborhood of the cases. Since the source population was explicitly unknown, the neighborhood sampling method was applied for this study. As a result, random selection was not possible during the time of the study. Upon detection of cases, we selected two unmatched controls for each Case (2 : 1) by using the neighborhood sampling method.

### 2.5. Sample Size Determination

The sample size was determined using EPI-info version 7, STATACALC software. The following assumption was applied, two side confidence level of 95% with a power of 80%. The percent of controls exposed was 30% to rove beetles, and the percent of cases with exposure was 49.1%. A minimum expected odds ratio of 2.25 was obtained from a Malaysian study [22]. Based on the above assumptions, the final sample size was 225 (75 cases and 150 controls).

### 2.6. Data Collection Tool and Procedure

A face-to-face interview was conducted using a structured questionnaire to collect the data on sociodemographic, potential personal behavior and environmental factors identified through the literature review. The questionnaire was written in English but the interviewer translated it into the local language (Amharic) orally while interviewing the study participants. The face and content validity of the questionnaire was assessed by experts and the relevant items under each domain were listed. These draft tools were sent to different health experts (2 epidemiologists and 1 dermatologist) for their opinion, taking into consideration the relevance of each variable to be retained in the tool. It was prepared using simple language, avoiding technical terms as much as possible to ensure accurate and reliable responses from the participants.

A questionnaire was prepared by reviewing different kinds of literature that were related to risk factors of Paederus dermatitis [1, 23]. It consisted of the following parts:

- Basic characteristics of participants: age, gender, region, marital status, educational level (illiterate, read and write, primary level, secondary level, or above secondary), place of residence, and number of a resident floor.
- Information on acute dermatitis and clinical feature (signs and symptoms of the illness, incubation period, site of the lesion, lesion feature, treatment, and outcome)
- Probable risk factors: personal behavior (sleep on the floor, wearing protective clothing, travel history to an endemic area, opening the window during the night, closed window before switching on the light, a habit of checking for wall or false ceiling (the upper interior surface of a room) before sleep, a habit of washing hand after contact with an irritant, use pesticide and repellant and bed net use), and environmental factor (surrounded by farmland, presence of rotten leaves, presence of outdoor light, and availability of window screen) [24, 25].

### 2.7. Data Quality Control

The questionnaire was pretested on 5% of the sample size in another district out of the study area before the actual data collection was implemented to ensure its acceptability and understandability, where required corrections and improvements were made following the pretesting process. Before the starting of data collection, a brief orientation was given to the data collectors. Each completed questionnaire was daily reviewed by the principal investigators to monitor the data quality. Before analysis, data were also cleaned for any missing and logically inconsistent values.

### 2.8. Data Analysis

Descriptive statistics including means and standard deviations of quantitative variables and frequencies (%) of qualitative variables were computed. Variables significantly (*p* ≤ 0.05) related to PD status on univariable logistic regression analysis were considered for their possible inclusion in the final multivariable conditional logistic regression model. The variables significantly (*p* < 0.05) and independently associated with PD status were retained in the final model. Multicollinearity was checked by taking >0.2 tolerance level. Model comparison was done using Bayesian information criterion (BIC) and Akaike's information criterion (AIC), Finally, the last model with the smallest value of the information criterion was selected as the final best fit model. Statistical analyses were performed using Statistical Package for the Social Sciences version 26.

### 2.9. Ethical Consideration

Ethical clearance was obtained from Ethiopian public health institutes, having a mandate for investigation of these public health emergencies [26]. Data collection was approved by the Addis Ababa Health Bureau after the submission of a formal request. Oral informed consent was obtained from all study participants, where the age was less than 16 years, assent was obtained from the children/adolescent, and permission was obtained from respective parents/guardians.

The research was undertaken in an emergency to respond to the outbreak immediately. The confidentiality of information was assured and ensured. Participants were treated with respect and willingly participated in the study with no payment or coercion. Besides, written informed consent was taken from the case to capture their lesions as a picture.

## 3. Results

### 3.1. Overall Description of the Outbreak

The first reports of a suspected PD case occurred on June 8^th^, 2018. There was a delay in reporting the suspected cases to the Ethiopian Public Health Institute, and, as a result, the investigation began on July 3^rd^, 2018. In total, 122 cases were reported by the subcity with no deaths.

**Figure 2:**
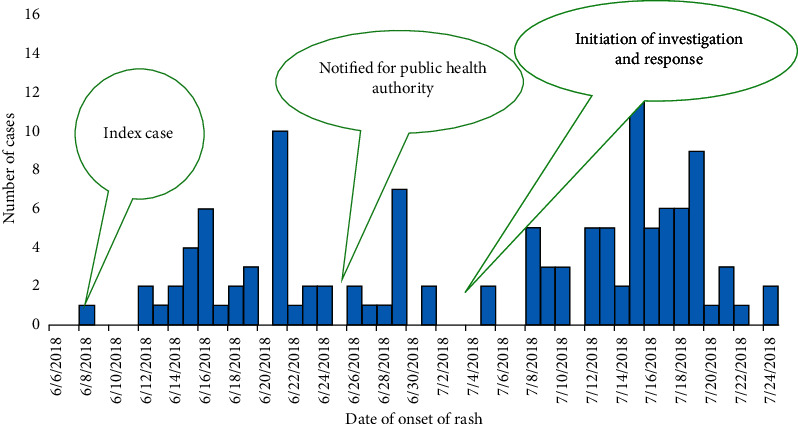
Epicurve of Paederus dermatitis outbreak in three districts of Bole subcity, Addis Ababa, Ethiopia, 2018.

**Figure 3:**
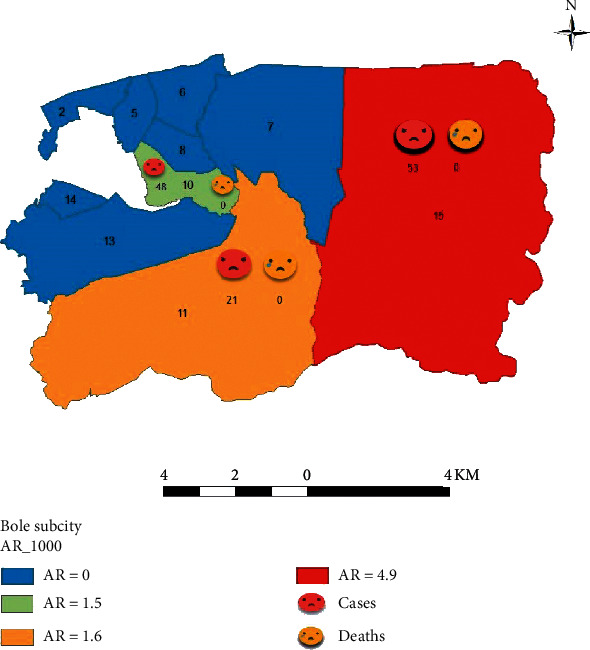
Spatial distribution of PD case along with attack rate in different district of Bole subcity, Addis Ababa, 2018.

During June, 74/122 cases (60.7%) were reported, while the remaining 48/122 (39.3%) cases were reported in July. Fifty-three (43.4%) cases were reported from District 15, followed by District 10, which reported 48 (39.3%), and District 11 reported 21(17.3). The total attack rate was 2.3/1000 population, with the highest attack rate in District 15 (4.9/1000) and the lowest in District 10 (1.5/1000). The epicurve of the outbreak and spatial distribution of the case are presented in Figures 2 and 3, respectively.

Cases were composed of 55 females (73.3%) and 20 males (26.7%), while the mean age of cases was (23.6 ± 16.4) with a minimum of 6 months and a maximum of 70 years. Controls were composed of 116 females (77.3%) and 34 males (22.7%). The mean age of controls was (29.4 ± 10.9) with a minimum of 10 years and with a maximum of 63 years (Table 1).

**Figure 4:**
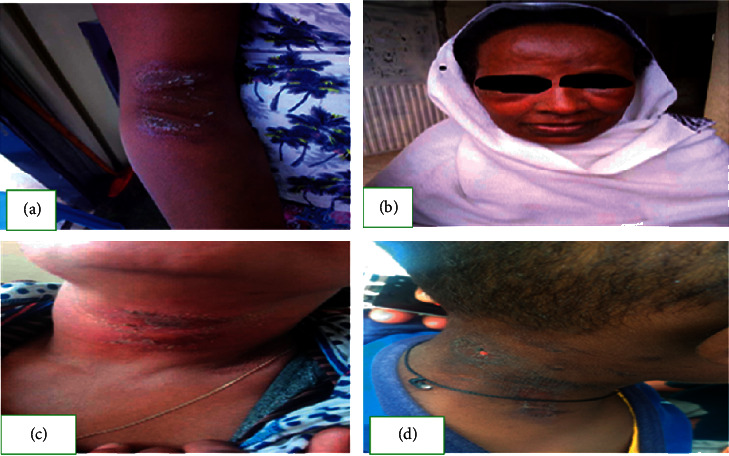
Pictures of some of the investigated cases in three districts of Bole subcity, Addis Ababa, Ethiopia, 2018. (a) Kissing lesion; (b) bilateral periorbital edema; (c, d) crusted plaque with healing erosion.

The incubation period was 1.9 ± 0.5 days after the contact with the beetle. Sixty (80%) cases presented with one lesion, 52 cases (69.3%) exhibited lesions on their faces, and all cases demonstrated erythema. Additionally, a pustule, erosion, and kissing lesion at the proximal part of the body were present in 18.7%, 50.7%, and 8.3% cases, respectively (Table 2 and Figure 4).

### 3.2. Risk Factor of Paederus Dermatitis

After applying both bivariate and multivariable logistic regression, only five variables had shown an overall significant effect on the risk of PD at the 5% level of significance (Table 3). In our multivariable analysis, the presence of outdoor light (AOR = 5.1; 95% CI (2.5, 10.9)), presence of rotten leaves in the surrounding area (AOR = 6.4; 95% CI (2.9, 15.7)), and sleeping on the floor (AOR = 6.1; 95% CI (2.5, 15.7)) were identified as risk factors prone to PD whereas the use of repellant (AOR = 0.1; 95% CI (0.0, 0.4)) and wearing protective clothing (AOR = 0.2; 95% CI (0.0, 0.4)) were identified as an independent protective factor.

## 4. Discussion

The outbreak of PD in the Bole subcity was characterized by an unusual upsurge in the population of *Paederus* species which resulted in increased cases of dermatitis in a different district of the subcity. Most cases were reported from district 15, which is surrounded by farmland and forest. This may be explained by urban expansion moving closer to agricultural fields and other sites where the beetles reside while rapid urbanization has the potential to reduce the risk of PD through the destruction of their natural habitat [1]; the prevalence of *Paederus* invasion into human settings has shown to be high [27].

The sociodemographic characteristics of the investigated cases revealed that most of the cases were female and age was below 15. The mean age of the cases was (22.9 ± 16.9) with a minimum of 6 months and with a maximum of 70 years. This reveals that all the sex and age groups are susceptible regardless of their social demographics character. The possibility of contracting dermatitis depends on personal behavior (remaining outdoors during the night, not wearing long-sleeve clothing) along with environmental exposure (surrounded by farmland) and the presence of the insect near to the human setting [16, 28–30].

The mean attack rate was 2.3/1000 population, the highest attack rate was in District 15, and the lowest attack rate was in District 10. The number of reported cases also varied from month to month, 39% cases reported during June and 61% reported during July. This designates that seasonality is one of the factors that determine the dispersion of the beetles. Previous studies conducted in India and Australia revealed that the incidence was during spring and summer and again during autumn and early winter. It indicates that the pattern may vary in different geographical and climatic zones of the country [5, 31, 32]. This may be explained by the food-seeking behavior of *Paederus* which begins when ambient temperatures rise above 20° [33, 34].

The incubation period ranges from one day to three days. This is consistent with studies conducted in Malaysia, Sri Lanka, and India, which showed the incubation period to last from one up to four days [12, 18, 35, 36].

The number of lesions determined by the frequency of contact with the insect or the number of insects the cases came into contact with the body. Sixty (80%) cases presented with one lesion and 12 (16%) cases presented with two lesions and the remaining cases presented with three lesions at a time. A study conducted in Iran revealed that most cases presented with multiple lesions (>3) at a time. In line with our findings, a study conducted in India showed that most cases presented with a single lesion at a time. The cases presented with itching, pain, burning sensation, and fever, which was a common sign and symptom in all cases. A hospital-based study in Sri Lanka shows that the most common symptom of cases was similar to our study [36].

Pederin results in a lesion in all parts of the body, except for the palm and sole. Our investigation found faces as the most common site which was consistent with previous findings in Iraq [11], Malaysia [22], and Sri Lanka [36]. This is explained by the uncovered part of the body being more vulnerable to the exposure of the beetles. The lesion features also differed from patient to patient, in which the manifestations comprise from erythema up to erosion including a kissing lesion. Kissing lesions related to the spread of pederin to adjacent skin on flexural surfaces [36]. A similar study conducted in Sri Lanka also revealed that most cases have similar lesion features, but, in some cases, regional lymphadenopathy was reported [36]. Similarly, a study in India found that the most common morphology of the lesion was erythema with that of vesicles and bullae [37, 38]. The morphology of the lesion is determined by the dose and duration of the toxin which contacts the skin [32, 39].

It has shown that *Paederus* species are vigorously attracted by artificial light and often tend to congregate around artificial light sources [31]. Our study revealed that the presence of outdoor light increases the risk of dermatitis. Comparative studies conducted in Sri Lanka [36], Turkey [9], India [26, 40], Malaysia [22], and Brazil [13] also revealed that artificial light contributes to an increase in the chances of contact with the beetle. The outdoor lights are attracting the insect to go inside. This commonly occurred in the inhabitant of humans and increased the chance of contact with beetle inadvertently [4, 17]. Additionally, beetles were found at a higher altitude to avoid impending obstacles, which, along with the light source, escalate the risk of contracting dermatitis [4]. The reduction of burning light and the use of mesh (net) at the light has a significant effect in reducing the chance of contact with beetles [36].

Our results show that the presence of rotten leaves has a strong association with dermatitis. The finding was comparable with a study conducted in Venezuela, Malaysia, and India [5, 25, 41]. Besides, the existence of farmland like vegetation, orange and lemon farms, and rice fields near to vicinity has a significant contribution to the outbreak. Furthermore, the presence of stagnant water, building construction and other such sites serve as potential harborage and breeding sites for the insect and their larvae.

This study was also able to identify that sleeping on the floor has an association with dermatitis. A study conducted in a closed population (military camp), as well as a nut farmer in Turkey, indicated that sleep on the floor is a possible risk factor for PD [5, 9]. This result can be explained by the insect's preference to run along the ground, despite their ability to fly [3, 42]. This study also found that wearing protective cloth (clothing with long sleeves) had a protective effect on dermatitis. This finding was in line with studies conducted in China and India [5, 34]. Uncovered part of the body has high level of susceptibility to dermatitis than the cover part of the body because it does not have a shade to prevent coincidental interaction with the insect [23].

According to this study, the use of repellent during the night was found protective against PD. This finding was in line with the study conducted in Angola and Malaysia [5]. Repellents like neem oil, coconut oil, and greasy ointments have a protective effect on dermatitis [30]. These repellents are thought to provides a protective layer from toxins that come in contact with our body [43, 44].

There were a few limitations to the present study. Firstly, recall bias is an inherent limitation of case-control study design, as cases might have remembered exposure to various risk factors better than the controls. Secondly, PD is not a usual outbreak in Ethiopia, due to the fact that this all-identified case should have to be included in the study for better identification of the factor that leads to the illness. Thirdly, an unbiased sampling and matched case-control method might be needed to improve the representativeness and efficiency of the study. Finally, the entomological investigation has limitations in determining the type of *Paederus* species responsible for the outbreak and vector density across the districts.

## 5. Conclusion

The study revealed that every individual, regardless of age or sex, predilection could develop dermatitis. This study showed that the presence of outdoor light, the presence of rotten leaves in the accommodation area, and sleeping on the floor were significantly associated with an increased PD risk, whereas wearing protective clothing and utilization of insect repellants offered significant protection against PD.

We recommended creating awareness of the condition among the local population and instructing them regarding the control measures. The measure should include reducing the outdoor light and removing excess decomposing plants and foliage from the accommodation site. Besides, simple preventive measures like avoidance of sleeping on the floor: dressing in appropriate cloth (long-sleeved tops and long trousers), and use of insect repellants during the night are advisable.

## Figures and Tables

**Table 1:** Sociodemographic characteristics of PD cases and controls, Addis Ababa, Ethiopia, 2018.

Sociodemographic characteristics	Case (%)	Control (%)
Sex		
Male	20 (26.7)	34 (22.7)
Female	55 (73.3)	116 (77.3)
Age group		
0–19	25 (33.3)	34 (22.7)
20–39	39 (52.9)	92 (61.3)
40–59	8 (10.7)	22 (14.7)
60–79	3 (4.0)	2 (1.3)
Ethnicity		
Oromo	24 (32.0)	40 (26.7)
Amhara	15 (20.0)	30 (20.0)
Tigrayans	21 (28.0)	42 (28.0)
Gurage	12 (16.0)	16 (10.7)
Others	3 (4.0)	22 (14.7)
Religion		
Orthodox	45 (60.0)	83 (55.3)
Muslim	15 (20.0)	28 (18.7)
Protestant	12 (16.0)	37 (24.7)
Catholic	3 (4.0)	2 (1.3)
Educational status		
Illiterate	6 (8.0)	0 (0.0)
Read and write	4 (5.3)	3 (2.0)
Primary	36 (48.0)	44 (29.3)
Secondary	18 (24.0)	61 (40.7)
Above secondary	11 (14.7)	42 (28.0)
Marital status		
Single	34 (45.3)	59 (39.3)
Married	34 (45.3)	80 (53.3)
Divorced	4 (5.4)	7 (4.7)
Window	3 (4.0)	4 (2.7)
Place of residence		
District (10)	28 (37.3)	48 (32.0)
District (11)	11 (14.7)	26 (17.3)
District (15)	36 (48.0)	76 (50.7)
Resident floor		
Ground floor	6 (8.0)	15 (10.0)
First floor	18 (24.0)	35 (23.3)
Second floor	7 (9.3)	17 (11.3)
Third floor	16 (21.3)	47 (31.3)
Fourth floor	28 (37.4)	36 (24.0)

**Table 2:** Frequency distribution of the illness characteristics among PD cases, Addis Ababa, Ethiopia, August 2018 (*N* = 75).

Characteristics of the illness	Frequency (*N* = 75)	Percent
Incubation period		
One day	10	13.3
Two days	59	78.9
Three days	6	8.0
Number of lesions		
One lesion	60	80.0
Two lesions	12	16.0
Three lesions	3	4.0
Sign and symptom		
Itching	74	98.7
Pain	72	96.0
Fever	6	8.0
Vomiting	0	0.0
Burning sensation	70	93.5
Site of lesion		
Face	52	69.3
Back	11	14.3
Back of neck	7	9.3
Shoulder	4	5.3
Lower limb	5	6.7
Upper limb	7	9.3
Lesion feature		
Erythema	75	100.0
Linear lesion	46	61.3
Vesicles	32	42.3
Pustules	14	18.7
Erosion	38	50.7
Kissing lesion	7	9.3
Treatment		
Topical steroid	30	40.0
Antibacterial	23	30.7
Antihistamine	11	14.7
Nonsteroid anti-inflammatory drug	6	8.0
Topical anesthesia	0	0.0
Local treatment	20	20.7
Complication		
Conjunctivitis	7	9.3
Temporal blindness	6	8.0
Residual pigmentation	24	32.0
Secondary infection	11	14.6

**Table 3:** Bivariate and multivariable logistic regression analysis result of Paederus dermatitis outbreak, Addis Ababa, Ethiopia August 2018.

Variable	Response	Cases (%)	Controls (%)	COR (95% CI)	AOR (95% CI)
Presence of outdoor light	Yes	53 (70.7)	43 (28.7)	6.0 (3.2–11.2)	5.1 (2.5–10.9)*∗*
	No_(rc)_	22 (29.3)	107 (71.3)	1	1
Presence of rotten leaves	Yes	61 (81.3)	57 (38.0)	4.5 (2.5–8.4)	6.4 (2.9–15.7)*∗*
	No_(rc)_	14 (18.7)	93 (62.0)	1	1
Sleeping on the floor	Yes	36 (48.0)	40 (25.8)	2.5 (1.4–4.6)	6.1 (2.5–15.7)*∗*
	No_(rc)_	39 (52.0)	110 (73.3)	1	1
Wearing protective clothing	Yes	42 (56.0)	108 (72.0)	0.5 (0.3–0.9)	0.2 (0.1–0.4)*∗*
	No_(rc)_	33 (44.0)	42 (28.0)	1	1
Using repellent	Yes	9 (12.0)	44 (29.3)	0.3 (0.1–0.6)	0.1 (0.0–0.4)*∗*
	No_(rc)_	66 (88.0)	106 (70.7)	1	1

rc = reference category; *n* = 225; *∗*variable is significant at *p* = 0.001.

## Data Availability

The datasets used and/or analyzed during the current study are available from the corresponding author on reasonable request.
